# Retraction: The Role of the Carbohydrate Recognition Domain of Placental Protein 13 (PP13) in Pregnancy Evaluated with Recombinant PP13 and the DelT_221_ PP13 Variant

**DOI:** 10.1371/journal.pone.0220517

**Published:** 2019-07-25

**Authors:** The PLOS ONE Editors

After publication of this article [1], concerns were raised about the following figures:

- In Figure 2B, there appear to be vertical discontinuities between lanes 1 and 2 of each panel, and the data reported in panels 3 and 4 appear similar. The authors provided raw image data from four replicate experiments, data from two of these were reported in the figure. The raw images clarified that the third and fourth panels in the figure errantly present data from the same replicate, and that lanes were removed from the original image between samples 1 and 2 of each panel and between the samples separated by black lines in the figure.
- There appears to be an image splicing line before the Elution 2 lane in Figure 2A.
- In Figure 3B, panel NRS (J0) and panel HRP (neg. control) appear similar when brightness and contrast are adjusted to visualize background details. The authors acknowledged there are similarities between these panels but assert that the images were obtained in different experiments.
- In Figure 4A, in panel 2, the 10 kDa DelT band and the 15 kDa nPP13 band appear to be the same, when the left side of the 10 kDa DelT band is slightly cropped.
- In Figure 4B, in panel 5, the 10 kDa DelT band and ~17 kDa rPP13 band appear to be the same, when the left side of the 10 kDa DelT band is slightly cropped.

The authors noted that the experiments were conducted when they were affiliated with Diagnostic Technologies, which provided support for aspects of the study. According to the authors, Diagnostic Technologies was dissolved prior to the article’s publication and the laboratory records and data for these experiments were destroyed during the company’s closure.

The underlying data for Figure 4 are not available. The authors provided supporting data to demonstrate that the antibodies recognize the three species of PP13 (wild type, native, truncated recombinant), but these did not address the image integrity concerns noted above and furthermore one of the images provided as negative control data for the confirmatory experiment did not contain verifiable image data.

In light of the unresolved concerns about Figures 3B and 4, the *PLOS ONE* Editors retract this article.

MS, BH, SG, HM did not agree with retraction. SN, RG, GO did not respond.

## References

[1] Sammar M, Nisamblatt S, Gonen R, Huppertz B, Gizurarson S, Osol G, et al (2014) The Role of the Carbohydrate Recognition Domain of Placental Protein 13 (PP13) in Pregnancy Evaluated with Recombinant PP13 and the DelT_221_ PP13 Variant. PLoS ONE 9(7): e102832 10.1371/journal.pone.0102832 25079598 PMC4117483

